# Ca-Alginate Hydrogel with Immobilized Callus Cells as a New Delivery System of Grape Seed Extract

**DOI:** 10.3390/gels9030256

**Published:** 2023-03-22

**Authors:** Elena Günter, Oxana Popeyko, Sergey Popov

**Affiliations:** Institute of Physiology of Federal Research Centre “Komi Science Centre of the Urals Branch of the Russian Academy of Sciences”, 50, Pervomaiskaya Str., 167982 Syktyvkar, Russia

**Keywords:** alginate, callus, cells, hydrogel, delivery system, grape seed extract

## Abstract

The development of new delivery systems for polyphenols is necessary to maintain their antioxidant activity and targeted delivery. The purpose of this investigation was to obtain alginate hydrogels with immobilized callus cells, in order to study the interaction between the physicochemical properties of hydrogels, texture, swelling behaviour, and grape seed extract (GSE) release in vitro. The inclusion of duckweed (LMC) and campion (SVC) callus cells in hydrogels led to a decrease in their porosity, gel strength, adhesiveness, and thermal stability, and an increase in the encapsulation efficiency compared with alginate hydrogel. The incorporation of LMC cells (0.17 g/mL), which were smaller, resulted in the formation of a stronger gel. The Fourier transform infrared analyses indicated the entrapment of GSE in the alginate hydrogel. Alginate/callus hydrogels had reduced swelling and GSE release in the simulated intestinal (SIF) and colonic (SCF) fluids due to their less porous structure and the retention of GSE in cells. Alginate/callus hydrogels gradually released GSE in SIF and SCF. The faster GSE release in SIF and SCF was associated with reduced gel strength and increased swelling of the hydrogels. LMC-1.0Alginate hydrogels with lower swelling, higher initial gel strength, and thermal stability released GSE more slowly in SIF and SCF. The GSE release was dependent on the content of SVC cells in 1.0% alginate hydrogels. The data obtained show that the addition of callus cells to the hydrogel provides them with physicochemical and textural properties that are useful for the development of drug delivery systems in the colon.

## 1. Introduction

Grape seed procyanidins have strong antioxidant properties [1,2]. They are able to protect against oxidative stress by reducing the concentration of free radicals, blocking their propagation and chelating metals [3]. Grape seed polyphenols are represented by monomers (catechin, epicatechin, epicatechin 3-O-gallate, gallocatechin, epigallocatechin), dimers, trimers, and polymerized procyanidins [4,5]. Grape seed extract (GSE) increases the number of colonic goblet cells, decreases the activity of colonic myeloperoxidase, and attenuates inflammation [3,6], as well as improving jejunal health by suppressing inflammation and regulating alkaline phosphatase [7]. Grape seed proanthocyanidins may modulate the gut microbiota by increasing levels of *Lactobacilli* and *Bacteroides*, thus representing an alternative approach to the treatment of IBD [3,6]. Proanthocyanidins can reduce the oxidation reaction in the intestine [8].

However, procyanidins are poorly soluble in water, unstable in the biological environment, and affected by temperature, pH, light, and relative humidity [2,3]. Grape seed procyanidins have low bioavailability due to their high molecular weight [9,10]. Therefore, the food and pharmaceutical industries cannot fully utilize these valuable compounds for their intended purpose. One approach to solving the problem of unstable phenolic compounds is to use a method of encapsulating these compounds, which protects phenols during transit in the gastrointestinal tract [11,12,13,14,15,16]. Grape seed proanthocyanidins were encapsulated in alginate–pectin microspheres [17], alginate–cellulose microcapsules [18], alginate–chitosan microparticles [19], alginate–oil–chitosan capsules [20], pectin– pullulan films [21], chitosan particles [22], chitosan–lecithin microspheres [23], *Bletilla striata* polysaccharide/chitosan microspheres [24], and phospholipid vesicles [8]. In most cases, the encapsulating agents cannot guarantee the targeted polyphenols release. In previous studies of the kinetics of polyphenol release from alginate–pectin beads, alginate beads and pectin films showed an increase in release (up to 90%) in the first 5–60 min, followed by a sustained release and then a plateau [17,25,26].

The development of new delivery systems for polyphenols is necessary to maintain their antioxidant activity and targeted delivery. Therefore, in the present study, the encapsulation of GSE was performed in alginate hydrogel matrices with immobilized cells of callus cultures. Alginate is a polysaccharide, which consists of residues of L-guluronic and D-mannuronic acids, forming hydrogels with the participation of divalent cations [27,28,29]. Alginate can protect GSE during gastric passage because it is stable in acidic environments. Alginate gel matrices mixed with other polymer matrices are capable of retaining polyphenols. It has been shown that alginate/cellulose microcapsules are able to significantly retain GSE at elevated temperatures and at a decreased pH, when compared to alginate microcapsules [18]. In recent years, hydrogels have been produced with immobilized plant cells, which can help create a unique food texture and expand the range of plant-based food production with valuable functional properties [30,31,32,33,34,35,36]. Callus cells immobilized in a hydrogel have the unique texture of artificial plant tissues, which are associated both with their special porous microstructure and cell turgor pressure [31,32]. In this study, we propose the use of callus cells immobilized in alginate hydrogel, since we assume that callus cells with a pore microstructure can facilitate the penetration and retention of GSE in the alginate/callus hydrogel, and then the gradual release of GSE in gastroenteric environments. Cells of campion and duckweed callus cultures were chosen as model cells for immobilization in alginate hydrogel, since they differ in size by a factor of two, which will have an effect on the properties and texture of the composite hydrogels and the ability to retain GSE in the hydrogel matrix. In addition, it is not known how the incorporation of callus cultures cells into an alginate hydrogel will alter the physicochemical and textural properties of the latter, or how it will alter the swelling and the ability to retain and release GSE.

The purpose of this investigation was to obtain alginate hydrogels with immobilized cells of different callus cultures, in order to evaluate their physicochemical characteristics and texture and to study the interaction between the physicochemical properties of hydrogels, texture, swelling behaviour, and grape seed extract release in vitro.

## 2. Results and Discussion

### 2.1. Characterization of Hydrogels Formed from Alginate and Cells of Different Callus Cultures

**Figure 1:**
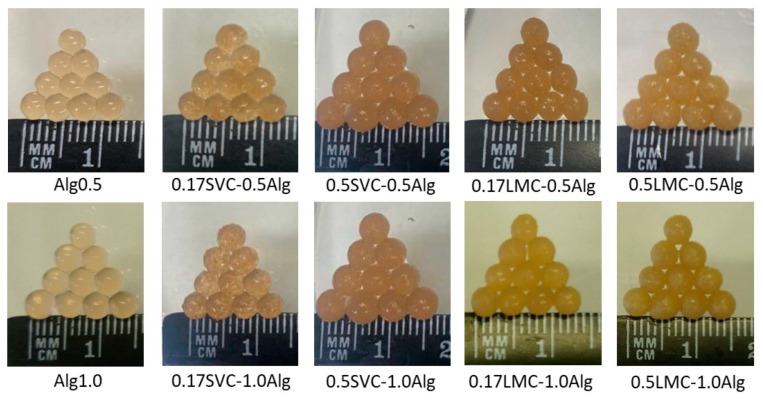
Digital images of GSE-loaded Ca-alginate particles and hydrogel particles based on alginate and cells of campion (SVC) and duckweed (LMC) callus cultures.

**Figure 2:**
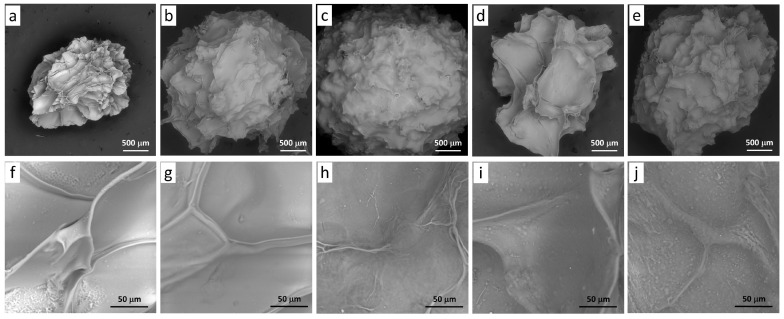
Scanning electron micrographs of GSE-loaded particles: Alg1.0 (**a**,**f**), 0.17SVC-1.0Alg (**b**,**g**), 0.5SVC-1.0Alg (**c**,**h**), 0.17LMC-1.0Alg (**d**,**i**), and 0.5LMC-1.0Alg (**e**,**j**). Magnification 63×, scale bar 500 μm (**a**–**e**) and magnification 948×, scale bar 50 μm (**f**–**j**).

Hydrogel particles of a spherical shape were obtained from sodium alginate (Alg, 0.5 and 1.0%), campion (SVC) and duckweed (LMC) callus cells (0.17, 0.33, and 0.50 g/mL), and GSE (1.3 mg/mL) using ionotropic cross-linking with 1.0% calcium lactate (Figure 1). The surface morphology of freeze-dried GSE-loaded Ca-alginate (Alg1.0) and Alg1.0/callus (0.17SVC-1.0Alg, 0.5SVC-1.0Alg, 0.17LMC-1.0Alg, 0.5LMC-1.0Alg) hydrogel particles is shown in Figure 2. All particles had a spherical shape and a rough and wrinkled surface, which could be due to the collapse of the hydrogel network after freeze-drying [37,38]. Alg1.0 particles had deep wrinkles and folds (Figure 2a) and a porous microstructure (Figure 2f). A wrinkled morphology has previously been shown for alginate capsules prepared from 1.0% alginate [18]. The surface roughness of gel beads was higher at low concentrations of sodium alginate [39] and alginate microspheres had a porous surface morphology [17]. The presence of callus cells had a significant effect on the morphological characteristics of the resulting hydrogels. Alg1.0/callus particles (Figure 2b–e) had shallower wrinkles and folds compared to Alg1.0 particles (Figure 2a). With an increase in the content of cells in hydrogels, a decrease in the number and depth of folds on the particle surface was noted (Figure 2b–e). In addition, a decrease in the thickness of wrinkles and folds was observed at a magnification of 948× (Figure 2g–j). Thus, a less rough surface was formed by increasing the ratio of cells to alginate. In addition, granular surface morphology was observed in particles based on LMC cells and alginate at a magnification of 948× (Figure 2i,j), which could be due to the smaller size of these callus cells compared to SVC cells (Figure 2g,h).

The diameters of Alg0.5 and Alg1.0 particles based on alginate alone were 3.303 and 3.631 mm, respectively (Table 1). The inclusion of callus cells caused an increase in the diameter of alginate beads in proportion to the concentration of callus cells. The diameter, gel strength, work, and elasticity of GSE-loaded hydrogels based on sodium alginate alone at a concentration of 1.0% (Alg1.0) were 1.1, 2.6, 3.3, and 1.4 times higher, respectively, than those of hydrogels based on sodium alginate at a concentration of 0.5% (Alg0.5) (Table 1 and Table 2). In this case, the adhesiveness of the hydrogels had similar values. The increase in gel strength and elasticity with increasing alginate concentration was probably due to the increased amount of alginate COO^−^ groups binding Ca^2+^ ions, which led to the emergence of more cross-links and the formation of a strong Ca-alginate gel [17,40].

The inclusion of callus cells into Alg0.5 hydrogel caused a decrease in the gel strength, work, and adhesiveness of hydrogels by 1.3–2.2, 1.1–1.5, and 1.1 times, respectively, when compared with a hydrogel based on alginate alone, which may be due to a loosening of the gel structure (Table 2). The same trend was shown when callus cells were included into Alg1.0 hydrogels. The decrease in gel strength, work, and adhesiveness of hydrogels was 1.5–2.2, 1.2–1.8, and 1.1–1.2 times, respectively. The elasticity of Alg0.5 and Alg1.0 hydrogels increased and did not change with the inclusion of callus cells. The gel strength, work, and elasticity of GSE-loaded Alg1.0 hydrogels with immobilized callus cells were higher than those of Alg0.5 hydrogels with immobilized callus cells, which was linked to the high ion-binding ability of alginate (Table 2). The adhesiveness of the hydrogels was similar.

The initial gel strength of GSE-loaded Alg0.5/callus and Alg1.0/callus hydrogels decreased slightly with an increase in the callus cells content from 0.17 to 0.50 g/mL. A negative correlation was found between the content of cells and the gel strength (R^2^ = −0.663, *p* < 0.05 for SVC-0.5Alg and LMC-0.5Alg hydrogels; R^2^ = −0.801, *p* < 0.05 for SVC-1.0Alg and LMC-1.0Alg hydrogels). Stronger hydrogels were formed when duckweed callus cells were included in their composition at a low concentration (0.17 g/mL), which was probably due to the smaller size of duckweed callus cells (66.9 ± 7.6 µm) compared to campion callus cells (111.6 ± 14.4 µm). The adhesiveness and elasticity of Alg0.5/callus hydrogels had similar values (0.018–0.019 N and 1.98–2.36 mm, respectively). The same trend was observed for the adhesiveness (0.016–0.018 N) and elasticity (2.62–2.83 mm) of Alg1.0/callus hydrogels.

The efficiency of GSE encapsulation in hydrogels based on alginate alone at a concentration of 0.5 and 1.0% was 48 and 51%, respectively. The inclusion of callus cells in the hydrogels led to an increase in the encapsulation efficiency, which was possibly related to the ability of callus cells to retain GSE. The efficiency of GSE encapsulation in Alg0.5 hydrogels containing SVC and LMC cells was 64–66% and 66–72%, respectively. The efficiency of GSE encapsulation in Alg1.0 hydrogels containing SVC and LMC cells was 72–77% and 65–76%, respectively. Correlation analysis established a positive correlation (R^2^ = 0.600, *p* < 0.05) between alginate concentration and the efficiency of GSE encapsulation. The degree of encapsulation increased with an increasing concentration of alginate in the hydrogels.

The lower degree of GSE encapsulation in hydrogels based on alginate alone (Alg0.5 and Alg1.0) was probably due to the presence of pores, while alginate hydrogels containing callus cells were less porous. The method of low-temperature physical sorption of nitrogen showed that the pore size of alginate/callus hydrogels was less than 2 nm. The specific surface area and pore volume of these hydrogels could not be measured due to the very small pore size. The specific surface area, volume, and radius of pores for Alg0.5 and Alg1.0 particles were 19.95 m^2^/g, 0.050 cm^3^/g, 5.037 nm and 24.50 m^2^/g, 0.048 cm^3^/g, 3.915 nm, respectively. The pores may facilitate the release of GSE during particle preparation. A decrease in the volume and size of pores was shown in proportion to the concentration of alginate (from 0.5 to 1.0%), which could probably lead to an increase in the efficiency of GSE encapsulation. It has previously been shown for alginate-carrageenan films that with an increase in the carrageenan content and the degree of cross-linking with Ca^2+^, the pore size decreased [41]. It is possible that callus cells prevented the formation of pores in alginate hydrogels during their preparation and, therefore, favored the retention of GSE. It has previously been shown that proanthocyanidin-loaded alginate/pectin microparticles had a smaller pore size than alginate microspheres [17]. Previous studies have shown the high encapsulation efficiency of proanthocyanidins in alginate/cellulose microcapsules (73–87%) [18], alginate/oil/chitosan capsules (58–88%) [20], and gelatin/pectic microparticles (85%) [42]. The low efficiency of encapsulation of proanthocyanidins (10–16%) was noted in alginate–pectin microspheres [17]. Thus, the efficiency of GSE encapsulation in alginate/callus hydrogels is high and comparable to that of previously developed proanthocyanidin encapsulation systems.

### 2.2. Fourier Transform Infrared Analyses (FTIR)

**Figure 3:**
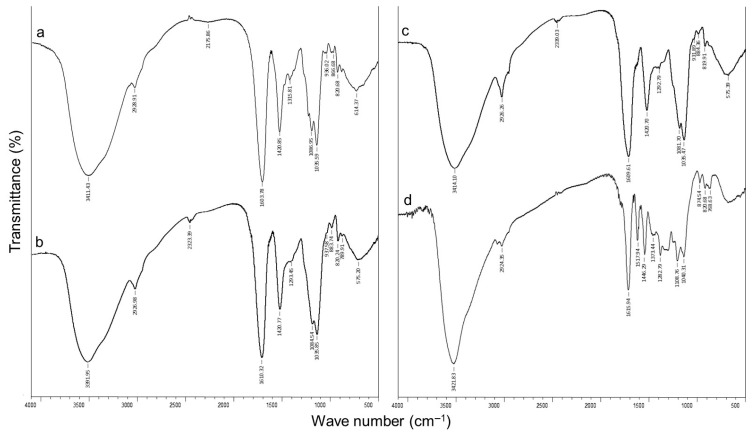
FTIR spectra of GSE-loaded Ca-alginate (Alg1.0) (**a**), alginate/callus particles (0.17SVC-1.0Alg and 0.17LMC-1.0Alg) (**b**,**c**), and GSE (**d**). GSE-grape seed extract.

The FTIR spectra of GSE-loaded Ca-alginate (Alg1.0), alginate/callus gels (0.17SVC-1.0Alg and 0.17LMC-1.0Alg), and GSE are presented in Figure 2. The spectrum of GSE-loaded Ca-alginate gels (Alg1.0) showed peaks at 3411, 2928, 1603, 1420, and 1035 cm^−1^, which correspond to the vibration of –OH, C–H, the asymmetric COO^−^, the symmetric COO^−^, and –C–O–C– groups, respectively [18,37,43,44,45,46] (Figure 3a). The bands at 1315, 1086, and 936 cm^−1^ indicate C–O stretching [38,43]. The FTIR spectra in the wave number COO^−^ of the Ca-alginate gels differed slightly from that of the sodium alginate powder, which was associated with cross-linking between Ca^2+^ and the COO^−^ groups of alginate [40,45].

The shift of the O–H (ca. 3400 cm^−1^) and COO^−^ (ca. 1600 cm^−1^) stretching vibrations was shown by comparing the FTIR spectra of the alginate/callus gels (0.17SVC-1.0Alg and 0.17LMC- 1.0Alg) with a spectrum of Ca-alginate gels (Alg1.0), which indicated the formation of hydrogen bonds in the network [17,25] (Figure 3b,c).

The GSE spectrum indicates a characteristic band of -OH stretching at 3422 cm^−1^, associated with the phenolic structure of GSE [18,42,47,48] (Figure 3d). The absorption peak at 2924 cm^−1^ indicates C–H stretching [18,42]. The absorption peaks at 1616, 1518, 1446, and 1283 cm^−1^ correspond to the aromatic ring [47,48]. The bands detected at 1616–1109 cm^−1^ could be attributed to the characteristic functional groups of the proanthocyanidins polyflavonoids [42,48]. The absorption peaks detected at 1373, 821, and 769 cm^−1^ are related to –C–OH deformation vibrations, the phenoxy substitution, and out-of-plane –CH deformation of aromatic rings, respectively [47,48].

The absorption peaks of GSE were mostly covered by the peaks of alginate hydrogel, indicating that GSE was embedded in the particles. It has previously been shown that the FTIR spectra of pectin-Zn-alginate particles loaded with GSE [40], pectin/pullulan films [21], alginate/cellulose derivatives microcapsules [18], and polysaccharide/chitosan microspheres [24] were similar to those of empty particles and films. Proanthocyanidins were physically encapsulated in a polymer matrix without chemical interaction with carrier materials [24]. Proanthocyanidins were entrapped in the matrix by hydrogen bonding or transformed into an amorphous structure [24]. The shift of the O–H stretching band (ca. 3400 cm^−1^) in the FTIR spectra of GSE-loaded Ca-alginate and alginate/callus gels compared to the FTIR spectrum of GSE may be due to the entrapment of GSE in the alginate hydrogel through hydrogen bonds (Figure 3).

### 2.3. Thermogravimetric Analysis (TGA) and Diffraction Scanning Calorimetry (DSC)

**Figure 4:**
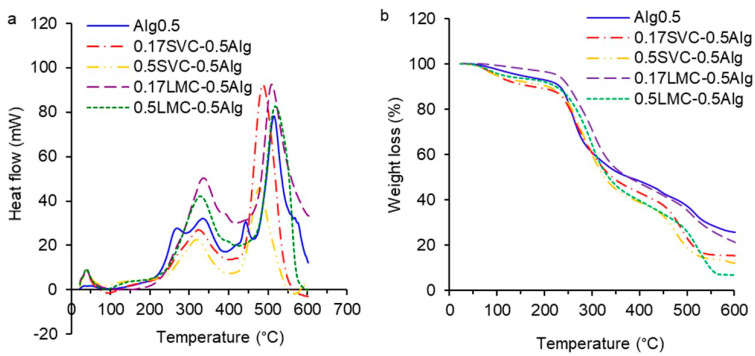
DSC (**a**) and TGA (**b**) thermograms of GSE-loaded Ca-alginate (Alg0.5) and Alg0.5/callus (0.17SVC-0.5Alg, 0.5SVC-0.5Alg, 0.17LMC-0.5Alg, 0.5LMC-0.5Alg) particles.

**Figure 5:**
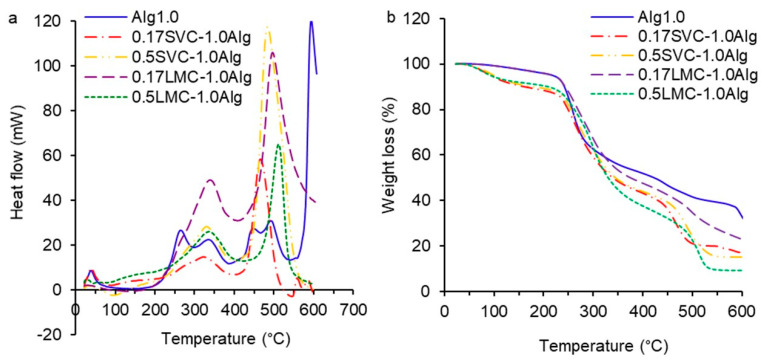
DSC (**a**) and TGA (**b**) thermograms of GSE-loaded Ca-alginate (Alg1.0) and Alg1.0/callus (0.17SVC-1.0Alg, 0.5SVC-1.0Alg, 0.17LMC-1.0Alg, 0.5LMC-1.0Alg) particles.

The DSC and TGA charts of GSE-loaded Ca-alginate (Alg0.5 and Alg1.0), Alg0.5/callus (0.17SVC-0.5Alg, 0.5SVC-0.5Alg, 0.17LMC-0.5Alg, 0.5LMC-0.5Alg), and Alg1.0/callus (0.17SVC-1.0Alg, 0.5SVC-1.0Alg, 0.17LMC-1.0Alg, 0.5LMC-1.0Alg) gels are presented in Figure 4 and Figure 5. On the DSC/TGA thermogram of the Alg0.5 hydrogel, a heat outflow peak was observed at 95.6 °C with a weight loss of 4.3%, which was associated with the loss of polymer-bound water (Figure 4a,b). The Alg0.5 hydrogel had exothermic peaks at 269.8 and 333.6 °C with an average weight loss of 40.5% and an exothermic peak at 443.3 °C with a weight loss of 3.5%, due to the alginate complex melting process [39]. Significant weight loss was associated with the degradation of the network between alginate and calcium, a rupture of the chains, and the depolymerisation of the alginate network structure [27,38]. An exothermic peak of Alg0.5 at 514.5 °C with a weight loss of 11.3% was due to the fragmentation of alginate into monomers, the complete thermal degradation of the polysaccharide, and the formation of carbon oxide [17,37].

The thermogram of the Alg1.0 hydrogel revealed exothermic peaks at 264.8 and 333.4 °C with an average weight loss of 41.2%, which were similar to those of the Alg0.5 hydrogel (Figure 5a,b). The Alg1.0 hydrogel had exothermic peaks at 448.3 and 493.5 °C and an average weight loss of 9.3%. The thermogram of the Alg1.0 hydrogel showed an exothermic peak at 594.2 °C and a weight loss of 7.3%. The shift of the last exothermic peak towards higher temperatures can be explained by an increase in the thermal stability of the hydrogel formed from the higher concentration of alginate as a result of the higher chelating strength between alginate and Ca^2+^ [39].

The DSC/TGA thermograms of 0.17SVC-0.5Alg and 0.5SVC-0.5Alg hydrogels exhibited endothermic peaks at 92.9 and 78.2 °C, respectively, due to water loss (Figure 4a). The 0.17SVC-0.5Alg and 0.5SVC-0.5Alg hydrogels showed similar exothermic peaks at 323.1 and 318.4 °C and weight loss of 39.5 and 43.3%, respectively (Figure 4a,b). The exothermic peaks of the 0.17SVC-0.5Alg and 0.5SVC-0.5Alg hydrogels at 487.0 and 477.4 °C were similar. The weight loss of these peaks was 23.6 and 21.5%, respectively. The degradation of the LMC-0.5Alg hydrogels and Ca-alginate hydrogel (Alg0.5) started at a higher temperature compared to the SVC-0.5Alg hydrogels. The 0.17LMC-0.5Alg and 0.5LMC-0.5Alg hydrogels had exothermic peaks at 336.1 and 327.3 °C with weight loss of 11.8 and 3.4%, respectively, as well as exothermic peaks at 509.2 and 518.1 °C with weight loss of 12.0 and 23.8%, respectively. In addition, exothermic peaks at 269.8 and 443.3 °C were absent in these alginate/callus hydrogels compared to the Ca-alginate hydrogel (Alg0.5), which was probably due to the presence of callus cells in the hydrogel composition.

On the DSC/TGA thermograms of SVC-1.0Alg and LMC-1.0Alg hydrogels, an endothermic peak at 79.1–95.1 °C with an average weight loss of 7–8% was observed due to dehydration (Figure 5a,b). The thermograms of 0.17SVC-1.0Alg, 0.5SVC-1.0Alg, 0.17LMC-1.0Alg, and 0.5LMC-1.0Alg hydrogels exhibited exothermic peaks at 321.7, 330.8, 339.1, and 337.2 °C and weight loss of 38.4, 40.6, 43.8, and 50.4%, respectively (Figure 5a,b). The 0.17SVC-1.0Alg, 0.5SVC-1.0Alg, 0.17LMC-1.0Alg, and 0.5LMC-1.0Alg hydrogels had exothermic peaks at 466.0, 483.9, 496.6, and 511.6 °C with weight loss of 19.8, 25.2, 14.1, and 19.8%, respectively. The destruction of LMC-1.0Alg hydrogels started at a higher temperature compared to SVC-1.0Alg hydrogels due to the increased thermal stability of LMC-1.0Alg hydrogels.

The destruction of SVC-1.0Alg and LMC-1.0Alg hydrogels started at a lower temperature compared with the Ca-alginate hydrogel (Alg1.0). The degradation of SVC-1.0Alg and LMC-1.0Alg hydrogels occurred faster, which was associated with the presence of callus cells in the composition of the alginate hydrogels, causing a loosening of the hydrogel structure. The loosening of the hydrogel structure is confirmed by a decrease in the strength of the gel and an increase in the content of callus cells (Table 2). Moreover, exothermic peaks at 264.8, 448.3, and 594.2 °C were absent in SVC-1.0Alg and LMC-1.0Alg hydrogels compared to the Alg1.0 hydrogel due to the presence of callus cells.

### 2.4. Swelling Behavior of GSE-Loaded Alginate Hydrogels with Immobilized Callus Cells

**Figure 6:**
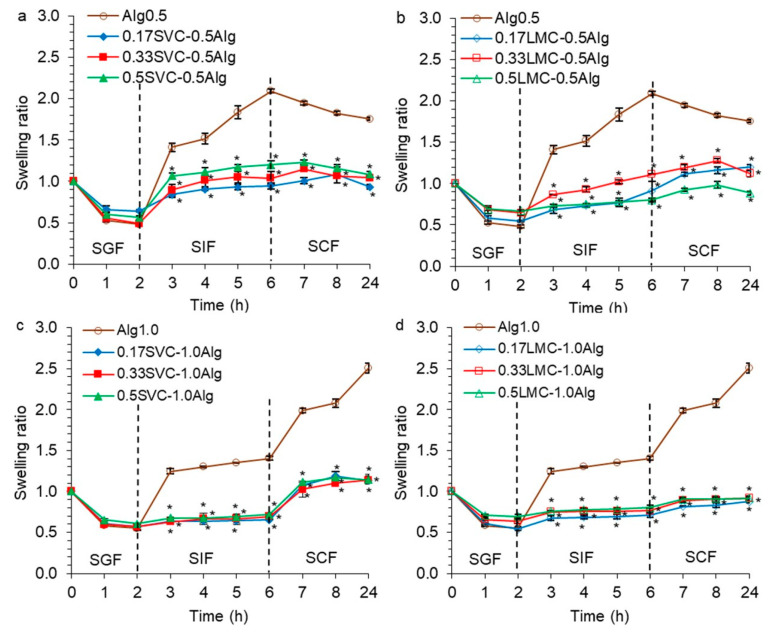
Swelling behavior of GSE-loaded hydrogels based on 0.5% alginate and SVC cells (**a**), 0.5% alginate and LMC cells (**b**), 1.0% alginate and SVC cells (**c**), 1.0% alginate and LMC cells (**d**) in the simulated gastric, intestinal, and colonic (SGF, SIF, and SCF, respectively) fluids. The data are presented as the mean ± S.D., *n* = 15. * *p* < 0.05 vs. 0.5 or 1.0% alginate.

It was found that all hydrogels loaded with GSE did not swell in the simulated gastric fluid (SGF, pH 1.25, 2 h), while the swelling ratio (SR) was 0.48–0.75 (Figure 6). The shrinkage of hydrogels in an acidic environment was observed. This phenomenon was due to the protonation of the carboxyl groups and a decrease in the electrostatic repulsion in an acidic environment [25,37,38,41]. In addition, it was shown that a decrease in the mobility of alginate chains and particle swelling was due to the formation of strong hydrogen bonds [15]. Our data are consistent with those of Tai et al. [19], who found that at gastric pH, shrinkage of the Ca-alginate gel caused a decrease in the size of alginate/chitosan microparticles. Shrinkage of repaglinide-loaded pectin–alginate particles was also noted at pH 1.2 [38]. Rayment et al. [49] found that alginate beads shrink in gastric fluid at pH 2.0 and swell in intestinal fluid at pH 8.0.

Ca-alginate hydrogels (Alg0.5 and Alg1.0) swelled rapidly in the first hour in SIF (pH 7.0) and gradually over the next 3 h (Figure 6a,c). The rapid swelling of the hydrogels in the SIF occurred due to the deprotonation of the alginate COO^−^ groups and their electrostatic repulsion, which led to the Ca-alginate network expansion and the fluid diffusion into the hydrogels [37]. A higher degree of swelling at intestinal pH than at stomach pH has also been shown previously for acacia gum–calcium alginate beads loaded with sodium diclofenac [37]. Higher swelling of the polyphenol-loaded alginate–pectin microspheres was also found at pH 6.0 compared to at pH 4.5 [25]. A slow swelling rate of pectin–alginate beads loaded with repaglinide was shown at pH 1.2 and faster swelling at pH 6.8 [38]. In SIF, Alg0.5 hydrogels (SR 1.4–2.1) swelled faster than Alg1.0 hydrogels (SR 1.2–1.4) due to the lower initial gel strength of Alg0.5 hydrogels as well as the larger pore volume and radius in Alg0.5 hydrogels, which led to the liquid diffusion and swelling. In SCF (pH 6.8 + pectinase, 18 h), Alg0.5 hydrogels decreased in size and degraded (SR 2.0–1.8), while Alg1.0 hydrogels continued to swell intensively (SR 2.0–2.5). Increased degradation of Alg0.5 hydrogels was associated with low initial gel strength (0.47 N) compared to Alg1.0 hydrogels (1.23 N) and the larger volume and size of pores. The data are confirmed by thermal analysis, which showed that Alg1.0 hydrogels had increased thermal stability compared to Alg0.5 hydrogels (Figure 4 and Figure 5). It has also been previously shown that beads with a lower alginate concentration swell faster than those with a higher concentration [43].

The inclusion of callus cells reduced the swelling of the alginate hydrogel in SIF and SCF by 2.0–2.4 and 1.9–2.9 times, respectively, compared with the swelling of a hydrogel based on alginate alone, which can be attributed to the less porous structure of alginate/callus hydrogels (Figure 6). In addition, the callus cells prevented the expansion of the alginate network. A change in the content of callus cells did not significantly affect the swelling of the hydrogels. The SVC-0.5Alg and LMC-0.5Alg hydrogels swelled slowly in SIF and then gradually degraded in SCF, which was similar to Alg0.5 hydrogels (Figure 6a,b). The SVC-1.0Alg and LMC-1.0Alg hydrogels gradually swelled in SIF and SCF (Figure 6c,d). The SVC-0.5Alg and LMC-0.5Alg hydrogels swelled faster (SR 0.7–1.2) than SVC-1.0Alg and LMC-1.0Alg hydrogels (SR 0.6–0.8) in SIF and then degraded in SCF, which was probably due to the low initial gel strength of the Alg0.5/callus hydrogels (0.21–0.36 N) compared to Alg1.0/callus hydrogels (0.56–0.81 N). SVC-1.0Alg hydrogels swelled faster in SCF (SR 1.0–1.2) than LMC-1.0Alg hydrogels (SR 0.8–0.9), which was probably due to the lower thermal stability and gel strength of SVC-1.0Alg hydrogels (Figure 6c,d).

### 2.5. The Release of GSE from Alginate Hydrogels with Immobilized Callus Cells

**Figure 7:**
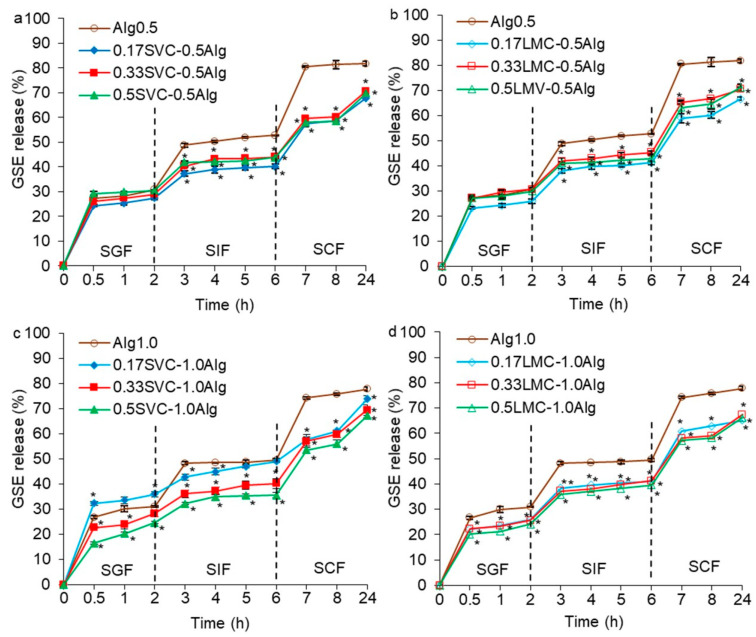
The release of GSE in vitro from hydrogels based on 0.5% alginate and SVC cells (**a**), 0.5% alginate and LMC cells (**b**), 1.0% alginate and SVC cells (**c**), 1.0% alginate and LMC cells (**d**) in the simulated gastric, intestinal, and colonic (SGF, SIF, and SCF, respectively) fluids. The data are presented as the mean ± S.D., *n* = 6. * *p* < 0.05 vs. 0.5 or 1.0% alginate.

The cumulative release of GSE from hydrogels in the simulated gastrointestinal fluids is presented in Figure 7. The GSE release in SGF was similar for all hydrogels, due to the very low swelling capacity of the hydrogels in an acidic fluid and increased shrinkage. A rapid GSE release was observed during the first 0.5 h of incubation (16–32%) followed by a very slow release over 1.5 h (24–36%).

Alg0.5 and Alg1.0 hydrogels released GSE more quickly in the first hour of incubation in SIF (48%) and slowly for 3 h (50–53%), which was consistent with the kinetics of hydrogel swelling. In SCF, Alg1.0 hydrogels released GSE more slowly (74–78%) than Alg0.5 hydrogels (81–82%). This could be due to the increased initial gel strength and thermal stability of Alg1.0 hydrogels, as well as the smaller pore size in hydrogels and the degradation of Alg0.5 hydrogels in SCF. The more intense release of GSE in SIF and SCF was associated with a decrease in the gel strength, which led to an increase in the diffusion of fluids into the hydrogels and leakage of GSE (Figure 7). The gel strength of Alg0.5 hydrogels decreased in SIF (4 h) and SCF (18 h) by 14.8 times compared with the initial gel strength. In SIF and SCF, the gel strength of Alg1.0 hydrogels decreased by 25.6 and 87.7 times, respectively. This may be due to the destruction of the cross-linking of the alginate network due to the replacement of Ca^2+^ by Na^+^ at pH 6.8 and 7.0 [18].

**Figure 8:**
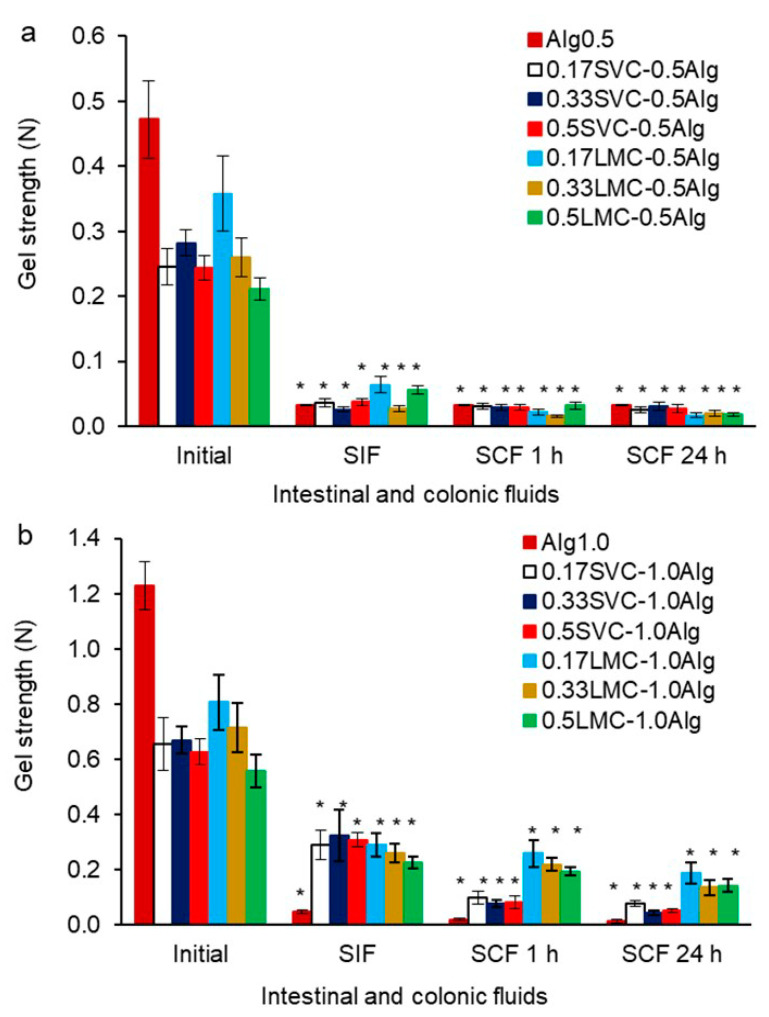
The gel strength of GSE-loaded hydrogels based on SVC and LMC cells and alginate at the concentration of 0.5% (**a**) and 1.0% (**b**) after successive exposure to SIF and SCF. The data are presented as the mean ± S.D., *n* = 15. * *p* < 0.05 vs. initial gel strength.

The addition of callus cells to alginate hydrogels led to a decrease in the release of GSE in SIF and SCF by 1.1–1.5 times compared with Ca-alginate hydrogels (Alg0.5 and Alg1.0) (Figure 7). This could be due to the reduced swelling capacity of alginate/callus hydrogels compared to Ca-alginate hydrogels due to the less porous structure of alginate/callus gels. Moreover, it is possible that GSE penetrated and was retained in callus cells and was then gradually released during the incubation in SIF and SCF. It has also been previously shown that alginate–cellulose microcapsules with less surface porosity than alginate microcapsules had a sustained release of GSE [18]. All alginate/callus hydrogels gradually released GSE in SIF and SCF. The faster GSE release was likely due to the reduction in gel strength after exposure to these fluids (Figure 8). The gel strength of alginate/SVC hydrogels decreased by 2.0–10.8 and 8.4–15.6 times in SIF and SCF, respectively. The gel strength of alginate/LMC hydrogels decreased by 2.4–9.6 and 3.9–21.1 times in SIF and SCF, respectively.

In previous studies regarding the kinetics of polyphenol release from alginate–pectin beads, alginate beads and pectin films at pH 6.0–7.4 showed an initial burst of release (40–90%) in the first 5–60 min, followed by a sustained release and a plateau [17,25,26]. In the present study, alginate/callus hydrogels released GSE gradually in SGF (16–36%), SIF (36–49%), and SCF (65–74%), indicating a controlled release of GSE in gastrointestinal media. At the same time, the GSE was released more quickly in SCF. This phenomenon could be due to lower swelling of alginate/callus hydrogels and high GSE retention capacity.

The release of GSE in SIF (40–45%) and SCF (67–71%) was similar for SVC-0.5Alg and LMC-0.5Alg hydrogels (Figure 7a,b). SVC-1.0Alg hydrogels released GSE in SIF and SCF slightly faster (36–49 and 67–74%) than LMC-1.0Alg hydrogels (40–41 and 65–67%) (Figure 7c,d). This could be attributed to the higher swelling of SVC-1.0Alg hydrogels, as well as a lower thermal stability and initial gel strength compared to LMC-1.0Alg hydrogels. In addition, the gel strength of SVC-1.0Alg hydrogels was significantly lower than that of the LMC-1.0Alg hydrogels after incubation in SCF (Figure 8b).

Alginate/callus hydrogels released a small amount of GSE (16–36%) in SGF comparable to the release of grape seed proanthocyanidins from alginate/cellulose capsules (21%) [18] and curcumin from alginate/ZnO beads (17%) [15]. At the same time, a significantly lower amount of GSE was released from alginate/callus hydrogels compared to that of grape seed proanthocyanidins from chitosan-based particles (88%) [22]. The smallest amount of GSE was released in SIF from 0.5SVC-1.0Alg (36%), 0.33SVC-1.0Alg (40%), 0.5LMC-1.0Alg (40%), 0.33LMC-1.0Alg (40%), 0.17LMC-1.0Alg (41%), 0.17SVC-0.5Alg (40%), and 0.17LMC-0.5Alg (41%) hydrogels. In our study, the release of GSE from alginate/callus hydrogels in SIF was lower than that of grape seed proanthocyanidins from chitosan (91%) [22] and alginate/cellulose (52%) particles [18]. Alginate/callus hydrogels released GSE in SIF in an amount similar to that of curcumin from alginate/ZnO beads (42%) [15]. Thus, the designed hydrogels have advantages over other proanthocyanidins delivery systems because they exhibit a controlled release in gastrointestinal environments.

Sheng et al. [18] also showed that an increase in the release of GSE from alginate microcapsules with an increase in pH from 2.0 to 10.0 was associated with the replacement of Ca^2+^ ions in the alginate hydrogel with Na+ ions from the sodium phosphate buffer. It has previously been shown that olive leaf polyphenols were released from alginate pectin beads faster at pH 6.0 than at pH 4.5 [25]. It was shown that the curcumin release from chitosan–pectin nanoparticles was negligible at pH 1.2, whereas it increased significantly in the medium with pectinase at pH 6.4 [12]. Curcumin was slightly released in SGF (pH 2.1) from the alginate/ZnO hydrogels, due to the low swelling ratio, and was rapidly released in SIF (pH 7.4) due to bead degradation [15]. The repaglinide release from pectin-alginate beads was higher at pH 6.8 than at pH 1.2, which was associated with swelling and diffusion processes [38].

The release profiles of GSE from SVC-0.5Alg, LMC-0.5Alg, and LMC-1.0Alg hydrogels did not depend on the content of cells in the hydrogels (Figure 7a,b,d). At the same time, the GSE release was dependent on the content of SVC cells in 1.0% alginate hydrogels (Figure 7c). A negative correlation (R^2^ = −0.722 − 0.994) was found between the content of SVC cells in the hydrogels and the amount of released GSE. Thus, there was a decrease in GSE release with an increase in the content of cells in SVC-1.0Alg hydrogels. This phenomenon was probably associated with such factors as the larger size of the SVC callus cells compared to the cells of the LMC callus and the higher concentration of alginate (1.0%). The high content of large SVC cells in the hydrogel enabled a greater retention of GSE when compared to hydrogels with a low content of such cells.

## 3. Conclusions

Alginate hydrogels with immobilized cells of different callus cultures (duckweed and campion) were produced. Differential scanning calorimetry, thermogravimetric analysis, Fourier transform infrared spectroscopy, and texture analysis were used to study the physicochemical characteristics of the hydrogels. The inclusion of callus cells in alginate hydrogels led to a decrease in the porosity, gel strength, adhesiveness, and thermal stability of the hydrogels compared with the hydrogels based on alginate alone, as well as to an increase in the encapsulation efficiency. A negative correlation was found between the content of callus cells in hydrogels and gel strength. The incorporation of LMC cells (0.17 g/mL), which were smaller, led to the formation of a stronger gel. The FTIR analyses indicated the entrapment of GSE in the alginate matrix. Alginate/callus hydrogels had reduced swelling and the GSE release in the simulated intestinal and colonic fluids, due to their less porous structure and the retention of GSE in cells. Alginate/callus hydrogels gradually released GSE in SIF and SCF, indicating a controlled release of GSE in gastrointestinal media. The faster GSE release in SIF and SCF compared to SGF was due to the reduction in gel strength and increased swelling. LMC-1.0Alg hydrogels with lower swelling, higher initial gel strength, and thermal stability released GSE more slowly in SIF and SCF. The GSE release was dependent on the content of SVC cells in 1.0% alginate hydrogels due to the large size of SVC cells and the higher concentration of alginate. In this study, we used, for the first time, an alginate hydrogel with immobilized callus cells as a system for delivering GSE to the colon. These hydrogels are capable of retaining and gradually releasing GSE in the gastrointestinal environment. The data obtained show that the addition of callus cells to the hydrogel provides them with physicochemical and textural properties that are useful for the development of drug delivery systems in the colon.

## 4. Materials and Methods

### 4.1. Materials

Sodium alginate was purchased from AppliChem, Germany. The grape seed extract (GSE) was obtained from Foodchem International Corporation, China. GSE contained 95% proanthocyanidins and 7.45% monomers (4.35% catechin and 3.10% epicatechin) according to data provided by the manufacturer. All other chemicals were of analytical grade. Callus cultures were taken from the collection of callus cultures of the Institute of Physiology of the Federal Research Center “Komi Science Center of the Urals Branch of the Russian Academy of Sciences”.

### 4.2. Callus Culture Cultivation

*Silene vulgaris* (Moench) Garcke (*Oberna behen* (L.) I.) and *Lemna minor* L. callus cultures were cultured on the solid modified Murashige and Skoog medium [50] in the darkness at 24 °C for 21 days. This medium included sucrose (15 g/L), glucose (15 g/L), agar (8 g/L), 2,4-dichlorophenoxyacetic acid (1.0–1.5 mg/L), and 6-benzylaminopurine (0.5 mg/L).

### 4.3. Development of Hydrogels and Their Characterization

Hydrogel particles were obtained from sodium alginate (0.5 and 1.0%), cells of callus cultures of duckweed (LMC) and campion (SVC) (0.17, 0.33, 0.50 g/mL), and GSE (1.3 mg/mL) in the presence of calcium lactate (1.0%). Sodium alginate (0.5 or 1.0%) was dissolved in distilled water, then 1 mL of GSE dissolved in ethanol (20 mg/mL) was poured into it. The final GSE concentration was 1.3 mg/mL. Callus cells of SVC or LMC were added to the resulting mixture at a concentration of 0.17, 0.33, and 0.50 g/mL and mixed. Hydrogel particles were obtained by extrusion of a mixture into a cross-linking solution of calcium lactate (1.0%) using a nozzle (inner diameter of 3.0 mm) and a distance of 5 cm. Then the hydrogels were exposed at 10 °C for 4 h and washed three times in distilled water. Alg0.5 and Alg1.0 particles without callus cells served as controls. Hydrogel particles formulations are shown in Table 1.

Projection equivalent diameter (diameter of circle with equivalent area) was measured for twenty wet particles of each sample. An optical microscope (Altami, Saint Petersburg, Russia) with a camera and an image analysis program (ImageJ 1.46r program, National Institutes of Health, Bethesda, Maryland, USA) were used to determine the particle diameter.

Micrographs of the surface morphology of freeze-dried GSE-loaded Ca-alginate and Alg1.0/callus gel particles were obtained using a scanning electron microscope (SEM) (Tescan Vega3 SBU, Brno, Czechia) at 20 kV and magnifications of 63× (scale bar 500 μm) and 948× (scale bar 50 μm).

The specific surface area, volume, and radius of pores of dry particles were measured by the low-temperature physical sorption of nitrogen using a Nova 1200e device (Quantachrome Instruments, Boynton Beach, USA). Before analysis, the hydrogels were freeze-dried using a Beta 2–8 LD plus (Martin Christ, Osterode am Harz, Germany) at the pressure of 0.021 mbar and the ice condenser temperature of −55 °C.

### 4.4. FTIR of GSE-Loaded Ca-Alginate and Alginate/Callus Particles

The FTIR spectra of GSE-loaded Ca-alginate (Alg1.0) and alginate/callus (0.17SVC-1.0Alg and 0.17LMC-1.0Alg) dry gel particles, and GSE were obtained using a FTIR spectrophotometer InfraLUM FT-08 (Lumex, Saint Petersburg, Russia). Before FTIR analysis, the hydrogels were freeze-dried using a Beta 2-8 LD plus (Martin Christ, Osterode am Harz, Germany). Each spectrum was the result of 20 scans and was obtained at 4000–400 cm^−1^ and a resolution of 4 cm^−1^.

### 4.5. The DSC and TGA Analysis

DSC and TGA analyzes of GSE-loaded Ca-alginate and alginate/callus dry gel particles were performed using a TGA/DSC3+ (Mettler Toledo, Greifensee, Switzerland). Before thermal analysis, the hydrogels were freeze-dried using a Beta 2–8 LD plus (Martin Christ, Osterode am Harz, Germany). Each sample (6–15 mg) was placed in a cuvette, and then heated at 25–600 °C (a heating rate of 10 °C/min).

### 4.6. Texture Analysis

The strength, work, adhesiveness, and elasticity of the hydrogels were measured on a Texture Analyzer (TA-XT Plus, Texture Technologies Corp., Stable Micro Systems, Godalming, UK). To assess the textural characteristics of wet hydrogels, the hydrogel particles were punctured using a P/2 probe. The probe movement speed was 1.0 mm/s. Each test was carried out for 15 particles. The gel strength, work, and elasticity of GSE-loaded hydrogels were determined using a Texture Exponent 6.1.4.0 software (Stable Micro Systems, Godalming, UK).

### 4.7. Swelling Study of Alginate Hydrogels with Immobilized Callus Cells

The wet hydrogel particles were exposed subsequently in simulated gastric fluid (SGF, pH 1.25) for 2 h, intestinal fluid (SIF, pH 7.0) for 4 h, and colonic fluid (SCF, pH 6.8 + pectinase) for 18 h. The SGF, SIF, and SCF were manufactured in accordance with [51,52] with modifications [53].

The wet hydrogel particles (1.2 g) were placed in swelling fluid (10 mL) and shaken at 37 °C and 100 rpm using an orbital shaker incubator (Titramax 1000, Heidolph, Schwabach, Germany). The particles were periodically collected and weighed. Excess moisture was soaked with filter paper and the particles were immediately weighed on an analytical balance. The swelling ratio (SR) of the particles was calculated using the following formula [43]: SR = W_t_/W_0_, where W_t_ is the weight of the particles after a determined incubation time in the fluid and W_0_ is the initial weight.

### 4.8. Calculation of Encapsulation Efficiency

The encapsulation efficiency was determined at 280 nm using a UV spectrophotometer (SEF-103, Akvilon, Moscow, Russia). The measurements were carried out in triplicate. The encapsulation efficiency (EE) was determined according to the equation: EE% = [(Q_t_ − Q_r_)/Q_t_] × 100, where Q_t_ is the amount of initial GSE and Q_r_ is the sum of the amount of GSE recovered in the aqueous solution after filtration and washing of particles.

### 4.9. The GSE Release In Vitro

The wet particles of each type (1.2 g) were sequentially incubated in 10 mL of solutions of SGF, SIF, and SCF for 2, 4, and 18 h, respectively. The samples were exposed at 37 °C and 100 rpm on a shaker (Titramax 1000, Heidolph, Schwabach, Germany). To study the GSE release from hydrocolloids, the aliquots were withdrawn at regular intervals and the absorbance was measured at 280 nm. To maintain a constant volume, measured aliquots were returned immediately. The experiments were performed three times.

### 4.10. Statistical Analysis

The mean ± standard deviation (S.D.) was used to present the results. The significance of differences between the two means was determined using Student’s *t*-test. At the same time, *p* < 0.05 was considered significant. Correlation analysis was performed to reveal the relationship between the studied characteristics of hydrogels. Statistica 10.0 software (StatSoft, Inc., Tulsa, OK, USA) and Excel Microsoft 2016 were used for statistical calculations.

## Figures and Tables

**Table 1:** Characterization of hydrogel particles.

Gel Formulation	Content of Callus Cells (g/mL)	Concentration of Alginate (%)	Diameter of Particles (mm)	Encapsulation Efficiency (%)
Alg0.5	0	0.5	3.303 ± 0.132	48.3 ± 0.2
0.17SVC-0.5Alg	0.17	0.5	3.759 ± 0.095 ^a^	63.6 ± 0.6 ^a^
0.33SVC-0.5Alg	0.33	0.5	3.907 ± 0.124 ^a^	67.8 ± 0.5 ^a^
0.5SVC-0.5Alg	0.50	0.5	4.056 ± 0.152 ^a^	65.8 ± 1.0 ^a^
0.17LMC-0.5Alg	0.17	0.5	3.688 ± 0.132 ^a^	72.4 ± 0.4 ^a^
0.33LMC-0.5Alg	0.33	0.5	3.811 ± 0.143 ^a^	68.9 ± 0.5 ^a^
0.5LMC-0.5Alg	0.50	0.5	3.933 ± 0.154 ^a^	65.5 ± 0.5 ^a^
Alg1.0	0	1.0	3.631 ± 0.122 ^a^	50.7 ± 1.0 ^a^
0.17SVC-1.0Alg	0.17	1.0	4.036 ± 0.143 ^a,b^	71.6 ± 0.3 ^a,b^
0.33SVC-1.0Alg	0.33	1.0	4.231 ± 0.161 ^a,b^	76.5 ± 0.3 ^a,b^
0.5SVC-1.0Alg	0.50	1.0	4.425 ± 0.178 ^a,b^	72.9 ± 0.3 ^a,b^
0.17LMC-1.0Alg	0.17	1.0	3.806 ± 0.152 ^a,b^	76.3 ± 0.5 ^a,b^
0.33LMC-1.0Alg	0.33	1.0	4.044 ± 0.128 ^a,b^	72.8 ± 0.4 ^a,b^
0.5LMC-1.0Alg	0.50	1.0	4.282 ± 0.104 ^a,b^	64.7 ± 0.3 ^a,b^

^a^ *p* < 0.05 vs. Alg0.5; ^b^ *p* < 0.05 vs. Alg1.0. The data are presented as the mean ± S.D., *n* = 20.

**Table 2:** Texture properties of hydrogels.

Gel Formulation	Initial Gel Strength (N)	Work (N·s)	Adhesiveness (N)	Elasticity (mm)
Alg0.5	0.472 ± 0.059	0.284 ± 0.034	0.020 ± 0.001	1.938 ± 0.075
0.17SVC-0.5Alg	0.246 ± 0.028 ^a^	0.192 ± 0.027 ^a^	0.019 ± 0.001 ^a^	1.977 ± 0.113
0.33SVC-0.5Alg	0.282 ± 0.020 ^a^	0.265 ± 0.039	0.019 ± 0.001 ^a^	2.329 ± 0.208 ^a^
0.5SVC-0.5Alg	0.244 ± 0.019 ^a^	0.242 ± 0.027 ^a^	0.018 ± 0.001 ^a^	2.362 ± 0.187 ^a^
0.17LMC-0.5Alg	0.358 ± 0.058 ^a^	0.251 ± 0.037 ^a^	0.018 ± 0.001 ^a^	1.992 ± 0.058 ^a^
0.33LMC-0.5Alg	0.260 ± 0.030 ^a^	0.251 ± 0.052 ^a^	0.018 ± 0.001 ^a^	2.225 ± 0.196 ^a^
0.5LMC-0.5Alg	0.211 ± 0.017 ^a^	0.377 ± 0.039 ^a^	0.018 ± 0.001 ^a^	2.351 ± 0.326 ^a^
Alg1.0	1.228 ± 0.086 ^a^	0.942 ± 0.079 ^a^	0.019 ± 0.004	2.678 ± 0.169 ^a^
0.17SVC-1.0Alg	0.654 ± 0.096 ^a,b^	0.626 ± 0.080 ^a,b^	0.018 ± 0.001 ^a^	2.679 ± 0.188 ^a^
0.33SVC-1.0Alg	0.670 ± 0.050 ^a,b^	0.678 ± 0.064 ^a,b^	0.017 ± 0.001 ^a^	2.709 ± 0.176 ^a^
0.5SVC-1.0Alg	0.575 ± 0.048 ^a,b^	0.537 ± 0.074 ^a,b^	0.016 ± 0.001 ^a,b^	2.607 ± 0.139 ^a^
0.17LMC-1.0Alg	0.808 ± 0.100 ^a,b^	0.761 ± 0.101 ^a,b^	0.017 ± 0.001 ^a^	2.776 ± 0.160 ^a^
0.33LMC-1.0Alg	0.715 ± 0.090 ^a,b^	0.789 ± 0.136 ^a,b^	0.016 ± 0.002 ^a,b^	2.829 ± 0.155 ^a,b^
0.5LMC-1.0Alg	0.558 ± 0.058 ^a,b^	0.574 ± 0.068 ^a,b^	0.016 ± 0.002 ^a,b^	2.618 ± 0.166 ^a^

^a^ *p* < 0.05 vs. Alg0.5; ^b^ *p* < 0.05 vs. Alg1.0. The data are presented as the mean ± S.D., *n* = 15.

## Data Availability

The data that support the findings of this manuscript are available from the corresponding author upon reasonable request.

## References

[1] Durazzo A., Lucarini M. (2019). Extractable and non-extractable antioxidants. Molecules.

[2] Zou Y.-C., Wu C.-L., Ma C.-F., He S., Brennand C.S., Yuan Y. (2019). Interactions of grape seed procyanidins with soy protein isolate: Contributing antioxidant and stability properties. LWT-Food Sci. Technol..

[3] Unusan N. (2020). Proanthocyanidins in grape seeds: An updated review of their health benefits and potential uses in the food industry. J. Funct. Foods.

[4] Shi J., Yu J., Pohorly J.E., Kakuda Y. (2003). Polyphenolics in grape seeds-biochemistry and functionality. J. Med. Food.

[5] Chedea V.S., Braicu C., Socaciu C. (2010). Antioxidant/prooxidant activity of a polyphenolic grape seed extract. Food Chem..

[6] Wang H., Xue Y., Zhang H., Huang Y., Yang G., Du M., Zhu M.J. (2013). Dietary grape seed extract ameliorates symptoms of inflammatory bowel disease in IL 10-deficient mice. Mol. Nutr. Food Res..

[7] Bibi S., Kang Y., Yang G., Zhu M.-J. (2016). Grape seed extract improves small intestinal health through suppressing inflammation and regulating alkaline phosphatase in IL-10-deficient mice. J. Funct. Foods.

[8] Manca M.L., Casula E., Marongiu F., Bacchetta G., Sarais G., Zaru M., Escribano-Ferrer E., Peris J.E., Usach I., Fais S. (2020). From waste to health: Sustainable exploitation of grape pomace seed extract to manufacture antioxidant, regenerative and prebiotic nanovesicles within circular economy. Sci. Rep..

[9] Goodrich K.M., Smithson A.T., Ickes A.K., Neilson A.P. (2015). Pan-colonic pharmacokinetics of catechins and procyanidins in male Sprague–Dawley rats. J. Nutr. Biochem..

[10] Fernández K., Roeckel M., Canales E., Dumont J. (2017). Modeling of the nnanoparticles absorption under a gastrointestinal simulated ambient condition. AAPS PharmSciTech..

[11] Fernandez K.F., Gonzalez M.A., Parada M.S. (2018). Transport of biodegradable polymeric particles loaded with grape seed extract across Caco-2 cell monolayers. Int. J. Food Sci. Technol..

[12] Alkhader E., Billa N., Roberts C.J. (2017). Mucoadhesive chitosan-pectinate nanoparticles for the delivery of curcumin to the colon. AAPS PharmSciTech..

[13] Tang D.-W., Yu S.-H., Ho Y.-C., Huang B.-Q., Tsai G.-J., Hsieh H.-Y., Sung H.-W., Mi F.-L. (2013). Characterization of tea catechins-loaded nanoparticles prepared from chitosan and an edible polypeptide. Food Hydrocoll..

[14] Li Z., Gu L. (2014). Fabrication of self-assembled (-)-epigallocatechin gallate (EGCG) ovalbumin–dextran conjugate nanoparticles and their transport across monolayers of human intestinal epithelial Caco-2 cells. J. Agric. Food Chem..

[15] Wang H., Gong X., Guo X., Liu C., Fan Y.-Y., Zhang J., Niu B., Li W. (2019). Characterization, release, and antioxidant activity of curcumin-loaded sodium alginate/ZnO hydrogel beads. Int. J. Biol. Macromol..

[16] Li Z., Ha J., Zou T., Gu L. (2014). Fabrication of coated bovine serum albumin (BSA)-epigallocatechin gallate (EGCG) nanoparticles and their transport across monolayers of human intestinal epithelial Caco-2 cells. Food Funct..

[17] Chen K., Zhang H. (2019). Alginate/pectin aerogel microspheres for controlled release of proanthocyanidins. Int. J. Biol. Macromol..

[18] Sheng K., Zhang G., Kong X., Wang J., Mu W., Wang Y. (2021). Encapsulation and characterisation of grape seed proanthocyanidin extract using sodium alginate and different cellulose derivatives. Int. J. Food Sci. Technol..

[19] Tie S., Su W., Zhang X., Chen Y., Zhao X., Tan M. (2021). pH-Responsive core-shell microparticles prepared by a microfluidic chip for the encapsulation and controlled release of procyanidins. J. Agric. Food Chem..

[20] Chen R., Guo X., Liu X., Cui H., Wang R., Han J. (2018). Formulation and statistical optimization of gastric floating alginate/oil/chitosan capsules loading procyanidins: In vitro and in vivo evaluations. Int. J. Biol. Macromol..

[21] Priyadarshi R., Riahi Z., Rhim J.-W. (2022). Antioxidant pectin/pullulan edible coating incorporated with Vitis vinifera grape seed extract for extending the shelf life of peanuts. Postharv. Biol. Technol..

[22] Muñoz V., Kappes T., Roeckel M., Vera J.C., Fernández K. (2016). Modification of chitosan to deliver grapes proanthocyanidins: Physicochemical and biological evaluation. LWT-Food Sci. Technol..

[23] Yu H.-L., Feng Z.-Q., Zhang J.-J., Wang Y.-H., Ding D.-J., Gao Y.-Y., Zhang W.-F. (2018). The evaluation of proanthocyanidins/chitosan/lecithin microspheres as sustained drug delivery system. BioMed Res. Int..

[24] Liu K., Feng Z., Shan L., Yang T., Qin M., Tang J., Zhang W. (2017). Preparation, characterization, and antioxidative activity of Bletilla striata polysaccharide/chitosan microspheres for oligomeric proanthocyanidins. Dry. Technol..

[25] Flamminii F., Di Mattia C.D., Nardella M., Chiarini M., Valbonetti L., Neri L., Difonzo G., Pittia P. (2020). Structuring alginate beads with different biopolymers for the development of functional ingredients loaded with olive leaves phenolic extract. Food Hydrocoll..

[26] Basanta M.F., Rojas A.M., Martinefski M.R., Tripodi V.P., De’Nobili M.D., Fissore E.N. (2018). Cherry (Prunus avium) phenolic compounds for antioxidant preservation at food interfaces. J. Food Eng..

[27] Fang Y., Al-Assaf S., Phillips G.O., Nishinari K., Funami T., Williams P.A. (2008). Binding behavior of calcium to polyuronates: Comparison of pectin with alginate. Carbohydr. Polym..

[28] Paques J.P., van der Linden E., van Rijn C.J.M., Sagis L.M.C. (2014). Preparation methods of alginate nanoparticles. Adv. Colloid. Interface. Sci..

[29] Gonçalves V.S.S., Gurikov P., Poejo J., Matias A.A., Heinrich S., Duarte C.M.M., Smirnova I. (2016). Alginate-based hybrid aerogel microparticles for mucosal drug delivery. Eur. J. Pharm. Biopharm..

[30] Seidel J., Ahlfeld T., Adolph M., Kümmritz S., Steingroewer J., Krujatz F., Bley T., Gelinsky M., Lode A. (2017). Green bioprinting: Extrusion-based fabrication of plant cell-laden biopolymer hydrogel scaffolds. Biofabrication.

[31] Vancauwenberghe V., Baiye Mfortaw Mbong V., Vanstreels E., Verboven P., Lammertyn J., Nicolai B. (2019). 3D printing of plant tissue for innovative food manufacturing: Encapsulation of alive plant cells into pectin based bio-ink. J. Food Eng..

[32] Park S.M., Kim H.W., Park H.J. (2020). Callus-based 3D printing for food exemplified with carrot tissues and its potential for innovative food production. J. Food Eng..

[33] Varma A., Gemeda H.B., McNulty M.J., McDonald K.A., Nandi S., Knipe J.M. (2021). Immobilization of transgenic plant cells towards bioprinting for production of a recombinant biodefense agent. Biotechnol. J..

[34] Landerneau S., Lemarié L., Marquette C., Petiot E. (2022). Green 3D bioprinting of plant cells: A new scope for 3D bioprinting. Bioprinting.

[35] Nordlund E., Lille M., Silventoinen P., Nygren H., Seppänen-Laakso T., Mikkelson A., Aura A.-M., Heiniö R.-L., Nohynek L., Puupponen-Pimiä R. (2018). Plant cells as food—A concept taking shape. Food Res. Int..

[36] Belova K., Dushina E., Popov S., Zlobin A., Martinson E., Vityazev F., Litvinets S. (2023). Enrichment of 3D-printed k-carrageenan food gel with callus tissue of narrow-leaved lupin Lupinus angustifolius. Gels.

[37] Benfattoum K., Haddadine N., Bouslah N., Benaboura A., Maincent P., Barillé R., Sapin-Minet A., El-Shall M.S. (2017). Formulation characterization and in vitro evaluation of acacia gum-calcium alginate beads for oral drug delivery systems. Polym. Adv. Technol..

[38] Awasthi R., Kulkarni G.T., Ramana M.V., de Jesus Andreoli Pinto T., Kikuchi I.S., Dal Molim Ghisleni D., de Souza Braga M., De Bank P., Dua K. (2017). Dual crosslinked pectin-alginate network as sustained release hydrophilic matrix for repaglinide. Int. J. Biol. Macromol..

[39] Aquino R.P., Auriemma G., D’Amore M., D’Ursi A.M., Mencherini T., Del Gaudio P. (2012). Piroxicam loaded alginate beads obtained by prilling/microwave tandem technique: Morphology and drug release. Carbohydr. Polym..

[40] Günter E.A., Popeyko O.V. (2022). Delivery system for grape seed extract based on biodegradable pectin-Zn-alginate gel particles. Int. J. Biol. Macromol..

[41] Roh Y.H., Shin C.S. (2006). Preparation and characterization of alginate-carrageenan complex films. J. Appl. Polym. Sci..

[42] De Souza V.B., Thomazini M., Echalar Barrientos M.A., Nalin C.M., Ferro-Furtado R., Genovese M.I., Favaro-Trindade C.S. (2018). Functional properties and encapsulation of a proanthocyanidin-rich cinnamon extract (Cinnamomum zeylanicum) by complex coacervation using gelatin and different polysaccharides. Food Hydrocoll..

[43] Belščak-Cvitanović A., Komes D., Karlović S., Djaković S., Špoljarić I., Mršić G., Ježek D. (2015). Improving the controlled delivery formulations of caffeine in alginate hydrogel beads combined with pectin, carrageenan, chitosan and psyllium. Food Chem..

[44] Khaksar R., Hosseini S.M., Hosseini H., Shojaee-Aliabadi S., Mohammadifar M.A., Mortazavian A.M., Javadi N.H.S., Komeily R. (2014). Nisin-loaded alginate-high methoxy pectin microparticles: Preparation and physicochemical characterization. Int. J. Food Sci. Technol..

[45] Arab M., Hosseini S.M., Nayebzadeh K., Khorshidian N., Yousefi M., Razavi S.H., Mortazavian A.M. (2019). Microencapsulation of microbial canthaxanthin with alginate and high methoxyl pectin and evaluation the release properties in neutral and acidic condition. Int. J. Biol. Macromol..

[46] Duan H., Lü S., Qin H., Gao C., Bai X., Wei Y., Wu X., Liu M., Zhang X., Liu Z. (2017). Co-delivery of zinc and 5-aminosalicylic acid from alginate/N-succinyl-chitosan blend microspheres for synergistic therapy of colitis. Int. J. Pharm..

[47] Fu C., Yang D., Peh W.Y.E., Lai S., Feng X., Yang H. (2015). Structure and antioxidant activities of proanthocyanidins from elephant apple (Dillenia indica Linn. ). J. Food Sci..

[48] De Freitas E.D., Lima B.M., Rosa P.C.P., da Silva M.G.C., Vieira M.G.A. (2019). Evaluation of proanthocyanidin-crosslinked sericin/alginate blend for ketoprofen extended release. Adv. Powder Technol..

[49] Rayment P., Wright P., Hoad C., Ciampi E., Haydock D., Gowland P., Butler M.F. (2009). Investigation of alginate beads for gastro-intestinal functionality, Part 1: In vitro characterization. Food Hydrocoll..

[50] Murashige T., Skoog S.A. (1962). Revised medium for rapid growth and bioassays with tobaco tissue cultures. Physiol. Plant..

[51] Chang K.L.B., Lin J. (2000). Swelling behavior and the release of protein from chitosan-pectin composite particles. Carbohydr. Polym..

[52] Gebara C., Chaves K.S., Ribeiro M.C.E., Souza F.N., Grosso C.R.F., Gigante M.L. (2013). Viability of Lactobacillus acidophilus La5 in pectin-whey protein microparticles during exposure to simulated gastrointestinal conditions. Food Res. Int..

[53] Günter E.A., Popeyko O.V. (2016). Calcium pectinate gel beads obtained from callus cultures pectins as promising systems for colon-targeted drug delivery. Carbohydr. Polym..

